# PDielec: The calculation of infrared and terahertz absorption for powdered crystals

**DOI:** 10.1002/jcc.24344

**Published:** 2016-04-13

**Authors:** John Kendrick, Andrew D. Burnett

**Affiliations:** ^1^ School of Life SciencesUniversity of BradfordBradfordBD7 1DPUnited Kingdom; ^2^ School of ChemistryUniversity of LeedsLeedsLS2 9JTUnited Kingdom; ^3^ School of Electronic and Electrical EngineeringUniversity of LeedsLeedsLS2 9JTUnited Kingdom

**Keywords:** infrared spectroscopy, terahertz spectroscopy, solid state DFT, permittivity, phonon, effective medium theory

## Abstract

The Python package PDielec is described, which calculates the infrared absorption characteristics of a crystalline material supported in a non‐absorbing medium. PDielec post processes solid‐state quantum mechanical and molecular mechanical calculations of the phonons and dielectric response of the crystalline material. Using an effective medium method, the package calculates the internal electric field arising from different particle morphologies and calculates the resulting shift in absorption frequency and intensity arising from the coupling between a phonon and the internal field. The theory of the approach is described, followed by a description of the implementation within PDielec. Finally, a section providing several examples of its application is given. © 2016 The Authors. Journal of Computational Chemistry Published by Wiley Periodicals, Inc.

## Introduction

The molecular and solid‐state quantum mechanical (QM) calculations of response properties such as the frequencies and intensities of infrared (IR) and terahertz (THz) radiation absorption have become generally available in many molecular and solid‐state computer programs. A common approach is to assume the harmonic approximation and calculate the mass weighted force constant matrix (for molecules) or the dynamical matrix at the gamma point (for periodic solids). Diagonalization of the matrix gives the frequencies for absorption and the normal modes (molecules) or phonon displacements (periodic solids). The calculation of the absorption intensity for each mode requires the calculation of the change in dipole moment caused by the displacement of the atoms for that mode. For solids where there is a large separation of charge, there can be a large coupling between a phonon mode and the internal field within a particle resulting from its morphology. This article describes the PDielec package, which is written in Python and post processes the output of solid state QM and molecular mechanics (MM) based codes such as VASP,^1^ CASTEP,^2^ and GULP^3^ to predict the infrared absorption of crystalline insulator materials whose crystal size is small compared with the wavelength of the absorbing radiation. The package is suited for the calculation of the complex, frequency dependent permittivity, and its associated absorption of infrared radiation for a finely ground crystalline material dispersed in a low loss dielectric medium such as KBr or polytetrafluoroethylene (PTFE). A particular feature of the program is its ability to take into account the constant permittivity of the supporting medium and the particle shape of the material of interest through an effective medium theory. This article outlines the theory used by the program and gives some examples of the application of the program for ionic and molecular materials.

## Theory

Equation (1) describes Beer–Lambert's law^4^ where *α* is the (decadic) absorption coefficient (usually given in cm^−1^), *I* and *I*_0_ are the intensities after and before absorption respectively and *d* is the path length.

(1) $$\begin{array}{c} \frac{I}{I_{0}}=10^{-\alpha d}\\ -\log\left(\frac{I}{I_{0}}\right)=\alpha d \end{array}$$

It is common, especially in the chemistry community, when reporting infrared spectra to use a decadic molar absorption coefficient (*a*), which has units of L mol^−1^ cm^−1^. The relationship between the absorption coefficient and the molar absorption coefficient^4^ is

(2) α=aC

where *C* is the concentration of the absorbing species.

### Molecular approach to absorption intensity

For molecules, the transition intensity *I_k_* of the *k* ^th^ mode (calculated from the change in dipole moment along the mode displacement) can be converted to an integrated molar absorption coefficient, *A_k_*, which can then be more readily compared with the experiment. The theory for this is described by Wilson et al.^5^ and results in expressions such as the two equations below [eq. (3)]. The first expression shows the relationship between the integrated molar absorption coefficient and the transition intensity and uses the number of molecules per unit volume (*N*), the velocity of light (*c*), and the degeneracy of the mode (*g_k_*). The second expression shows the appropriate conversion factors if the units for the integrated molar absorption coefficient are L mol^−1^ cm^−2^ (1 L mol^−1^ cm^−2^ = 0.01 km mol^−1^) and the units for the transition intensity are D^2^ Å^−2^ amu^−1^, where D represents the Debye unit of dipole moment and amu is an atomic mass unit. The factor log_e_10 arises due to the choice of a decadic Beer's law.

(3) $$\begin{array}{c} A_{k}=\frac{N\pi }{3c^{2}{\;\log}_{e}10}g_{k}I_{k}\\ A_{k}=\frac{N_{a}\pi }{3000c^{2}2.302585}g_{k}I_{k}=\text{4225.6}g_{k}I_{k} \end{array}$$

The derivation of the above expressions assumes that the rotational levels are not quantized and that the vibrational levels are thermally occupied according to a Boltzmann distribution. In order to use the calculated molecular intensities to predict a spectrum it is usual to assume^5^ that each transition is associated with a Lorentzian line shape with a full width at half maximum (FWHM) of *σ_k_*. It is common, when reporting comparison between theoretical and experimental spectra, to assume that the line widths are the same for all modes.^6^, ^7^ Recent work on terahertz absorption in crystalline pentaerythritol tetranitrate (PETN) using molecular dynamics calculations^8^ in combination with the direct calculation of the cubic anharmonic couplings of the normal modes^9^ has shown that the FWHM of the intense absorptions may vary between 10 and 25 cm^−1^. Assuming a Lorentzian line shape, the molar absorption coefficient for the *k*th mode at wavenumber, ${\bar{\nu }}_{k}$, can be written as a function of frequency or wavenumber ( $\bar{\nu }$);

(4) $$\begin{array}{c} a_{k}(\bar{\nu })=\frac{2A_{k}}{\pi }\frac{\sigma_{k}}{4{\left(\bar{\nu }-{\bar{\nu }}_{k}\right)}^{2}+\sigma_{k}^{2}}\\ a_{k}^{\max}=\frac{2A_{k}}{\pi \sigma_{k}} \end{array}$$

The maximum height of the Lorentzian, $a_{k}^{\max}$, clearly depends upon the value of *σ_k_*. As can be seen in eq. (5), the choice of normalization for the Lorentzian means that integration of the molar absorption coefficient over wavenumber returns the integrated molar absorption coefficient and a sum over all the bands provides the total molar absorption coefficient *a* ^mol^ ( $\bar{\nu }$) as a function of wavenumber, calculated from the intensities of each band. The final expression in eq. (5) shows the relationship between the absorption and the molar absorption coefficients. *C* is the concentration usually expressed in mol/L.

(5) $$\begin{array}{c} A_{k}=\int a_{k}(\bar{\nu })d\bar{\nu }\\ a^{\text{mol}}(\bar{\nu })=\sum_{k}a_{k}(\bar{\nu })\\ \alpha^{\text{mol}}(\bar{\nu })=Ca^{\text{mol}}(\bar{\nu }) \end{array}$$

A comment should be made about the various units which can be used for these quantities. A common unit for the transition intensity is (D/Å)^2^/amu, another is km/mol. However, it should be pointed out that strictly speaking the latter unit refers to the integrated molar absorption coefficient as defined above in eq. (3) and therefore relies on the assumptions made in its derivation (1 (D/Å)^2^/amu is equivalent to 42.256 km/mol).

### Solid‐state approach to absorption intensity

The optical properties of a solid are determined by its complex, frequency dependent relative permittivity ( $\bar{\bar{\varepsilon }}(\bar{\nu })$) and in particular the imaginary refractive index component tensor, $\bar{\bar{\kappa }}$, of the complex refractive index, $\bar{\bar{N}}$ where;

(6) $$\begin{array}{l} \bar{\bar{N}}{\left(\bar{\nu }\right)}^{2}=\bar{\bar{\varepsilon }}(\bar{\nu })\\ \bar{\bar{N}}(\bar{\nu })=\bar{\bar{n}}(\bar{\nu })+i\bar{\bar{\kappa }}(\bar{\nu }) \end{array}$$

The intensity of absorption is given by the effect of the imaginary component of the refractive index on the incident light assuming an isotropic material^6^

(7) $$\begin{array}{c} I=I_{0}e^{-4\pi \kappa (\bar{\nu })d/\lambda }\\ I=I_{0}e^{-4\pi \bar{\nu }\kappa (\bar{\nu })d}\\ -\ln\left(\frac{I}{I_{0}}\right)=4\pi \bar{\nu }\kappa (\bar{\nu })d\\ -\log\left(\frac{I}{I_{0}}\right)=4\pi \bar{\nu }\kappa (\bar{\nu })d.\log e \end{array}$$

Comparison with the definition of the absorption coefficient from Beer–Lambert's law [eq. (1)] gives

(8) $$\begin{array}{c} \alpha^{\text{sol}}(\bar{\nu })=4\pi \bar{\nu }\kappa (\bar{\nu })\cdot \log e\\ a^{\text{sol}}(\bar{\nu })=\frac{\alpha^{\text{sol}}(\bar{\nu })}{C} \end{array}$$

Since the refractive index is dimensionless, the absorption coefficient (*α* ^sol^) is specified in cm^−1^. The superscripts “sol,” for solid, and “mol,” for molecular, are used here to distinguish between the two methods of calculating the absorption (*α*) and molar absorption coefficients (*a*). In the calculation of the imaginary component of the refractive index it is necessary to choose the solution which gives a positive value. This is consistent with the Kramers–Kronig relationship between the real and imaginary components.^10^

In order to calculate the relationship between absorption and molar absorption coefficients it is necessary to know the concentration. For solid‐state calculations the required unit is moles of unit cells per liter. One of the drawbacks of this molar absorption coefficient unit is that the number of molecules in a unit cell can change depending on whether a supercell, primitive or non primitive unit cell is being used. A more natural unit would be to use a mole of formula units, or a mole of molecules. However for the rest of this paper eq. (9) will be used, where *V* is the volume of the unit cell, and therefore the concentration *C* is moles of unit cell/liter.

(9) $$C=\frac{f\cdot 1000\;{\text{ cm}}^{3}}{VN_{a}}$$

The volume fraction, *f*, of the dielectric material in a supporting matrix of non‐absorbing material is included in the expression for the concentration as it will be useful when the theory for mixtures is developed.

For a periodic system, the permittivity tensor can be calculated as a sum over Lorentz oscillators, incorporating an imaginary loss component through the damping factor *σ_k_*.^11^ The frequencies of the oscillators are the transverse optic (TO) phonon frequencies of the system.

(10a) $$\bar{\bar{\varepsilon }}(\nu )={\bar{\bar{\varepsilon }}}_{\infty }+\frac{4\pi }{V}\sum_{k}\frac{{\bar{\bar{S}}}_{k}}{\nu_{k}^{2}-\nu^{2}-i\sigma_{k}\nu }$$

(10b) $${\bar{\bar{S}}}_{k}={\bar{Z}}_{k}{\bar{Z}}_{k}^{T}$$

(10c) $${\bar{Z}}_{k}=\sum_{a}{\bar{\bar{Z}}}^{a}\;{\bar{U}}_{k}^{a}$$

(10d) $$\bar{\bar{D}}\;{\bar{U}}_{k}=\Lambda_{k}{\bar{U}}_{k}$$

(10e) $$\nu_{k}^{2}=\Lambda_{k}$$

*V* is the volume of the unit cell, ${\bar{\bar{S}}}_{k}$ is the dipole oscillator strength tensor for the *k*th transition, with a TO frequency of *ν_k_* and ${\bar{\bar{\varepsilon }}}_{\infty }$ the optical permittivity tensor, which represents the electronic contribution to the permittivity. The intensity of a transition, *I_k_* is given by the trace of the oscillator strength tensor, *I_k_* = *tr* ( ${\bar{\bar{S}}}_{k}$) . The damping factor *σ_k_* removes any discontinuities at the TO frequencies. Since the oscillator strengths and phonon frequencies can be calculated routinely in solid‐state QM packages, the calculation of the frequency‐dependent complex permittivity using eq. (10a) is straightforward. In some cases, using eqs. (10b) and (10c), PDielec calculates the oscillator strengths from the Born charge matrix for atom *a,* ${\bar{\bar{Z}}}^{a}$, and the contribution of atom *a* to the *k* ^th^ phonon mode, ${\bar{U}}_{k}^{a}$.^11^ As shown in eq. (10d), at the Γ point the *k* ^th^ phonon mode is described by the eigenvector, ${\bar{U}}_{k}$ , and eigenvalue, $\Lambda_{k}$ , of the mass weighted, dynamical matrix, $\bar{\bar{D}}$, which is a 3*N*×3*N* matrix, where *N* is the number of atoms in the unit cell. The eigenvalues are the squared frequencies of the phonon modes [eq. (10e)]. The displacement of each atom in the *k*th mode, is proportional to ${\bar{U}}_{k}^{a}/\sqrt{m_{a}}$ , where *m_a_* is the mass of atom *a*. The dynamical matrix has 3*N* eigenvectors and eigenvalues, of which three should be zero due to translational invariance. If there are any negative eigenvalues the system is unstable to some displacement and therefore not at an energy minimum.

For ionic systems it is common practice in solid‐state QM and MM programs to include a long wave‐length, non‐analytic correction to the mass weighted dynamical matrix at the Γ point, which describes the coupling of the longitudinal optic (LO) modes to the induced field resulting from the vibration. This may be written for atoms *s* and *t* and their Cartesian components *α* and *β* as^11^

(11) $${\left({\bar{\bar{D}}}_{q\to 0}^{\mathrm{LO}}\right)}_{\mathrm{s,\alpha ;t,\beta}}={\left(\bar{\bar{D}}\right)}_{\mathrm{s,\alpha ;t,\beta}}+\frac{4\pi }{V\sqrt{M_{s}M_{t}}}\frac{{\left({\bar{q}}^{T}{\bar{\bar{Z}}}_{s}\right)}_{\alpha }{\left({\bar{q}}^{T}{\bar{\bar{Z}}}_{t}\right)}_{\beta }}{{\bar{q}}^{T}\cdot \bar{\bar{\varepsilon }}\cdot \bar{q}}$$

The mass weighting has been incorporated through the mass of the atoms, *M*_s_ and *M*_t_. The correction depends upon the direction, $\bar{q}$, that the long wavelength limit is approached. Diagonalization of the corrected matrix gives the squared frequencies of *N* − 1 LO modes and 2*N* − 2 TO modes [eqs. (10d) and (10e)]. In some of the examples given below, the LO frequencies will be given for comparison with the TO frequencies.

### Effect of particle shape on infrared absorption

It has long been recognized that, especially for ionic materials, the local field within a crystal and its coupling with the transverse optic phonons has an important effect on the position and intensity of the absorption. Fröhlich^12^ was one of the first to point out that the frequency of absorption of a small ionic sphere embedded in a low dielectric medium is shifted to lie between the transverse and longitudinal optic frequencies of the material making up the sphere.

In the development of the theory used in PDielec, an important assumption is that the particle size of the crystallites in the sample is small compared with the wavelength of light. Using this approach, Genzel and Martin^13^ were able to explain the observed infrared absorption of small spheres of MgO crystallites and the effect of the permittivity of the supporting medium on the spectrum. Studies of the infrared absorption by small particles of α‐Fe_2_O_3_ using an effective medium theory and an absorption/scattering theory^14^, ^15^ showed that in order to fit the experimental spectra it was necessary to adjust not only the damping factors in eq. (10a), but also the permittivity of the matrix and the volume fraction of the dielectric medium. The latter was used to account for aggregation effects as the volume fraction increased. It was also shown that effective medium theories were only applicable for particles smaller than the wavelength of light. For larger particles the scattering from the particles becomes increasingly important.

More recently, Balan and coworkers in a series of papers^16^, ^17^, ^18^, ^19^ used density functional calculations together with an effective medium theory to calculate the infrared absorption of several minerals incorporating information about the crystallite shape. In an experimental and theoretical study of irradiated kaolinite,^19^ it was shown that exposure to radiation resulted in shifts in the infrared spectrum which could be accounted for by increasing the polarizability of the particles through an increase in the optical permittivity tensor.

The underlying theory adopted by PDielec is based on similar premises to the work described above, namely that the dielectric response of small spherical, ellipsoidal, slab‐like or needle‐like crystallites randomly distributed in a non‐absorbing medium such as PTFE, KBr, or Nujol is the same as that of an effective medium material whose frequency‐dependent dielectric response can be calculated from the frequency‐dependent permittivity tensor of the crystal (as calculated by solid state QM or MM calculations), the shape of the crystallites, and the permittivity of the non‐absorbing medium (taken to be a constant over the frequency range of interest).

The development of the theory reported here closely follows the work by Sihvola.^20^ It will be assumed that the inclusion particles, which may be non‐isotropic, ellipsoidal (including spherical, needle‐like, and plate‐like), are randomly orientated in an embedding, non‐absorbing medium such as PTFE, KBr, or Nujol. It should be emphasized that while PDielec can take account of particle shape, particle and matrix permittivity, there are many additional aspects of infrared absorption which need to be considered when comparing calculated and experimental results. Most notable of these are the coupling between phonons and mobile electrons or holes (so called phonon–polariton coupling),^21^ the scattering which starts to dominate as the particles get larger^19^ and the agglomeration of particles as the volume fraction increases.

### The polarizability of an isolated particle

**Figure 1:**
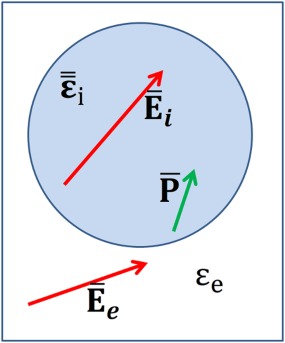
Schematic showing the field and polarization inside an inclusion with non‐isotropic permittivity embedded in a supporting medium. [Color figure can be viewed in the online issue, which is available at wileyonlinelibrary.com.]

Figure 1 shows a schematic of the field and polarization inside an inclusion with non‐isotropic permittivity ${\bar{\bar{\varepsilon }}}_{i}$ embedded in a supporting medium with permittivity *ε*_e_. The internal field within the inclusion is indicated by ${\bar{E}}_{i}$, the external, applied field is indicated by ${\bar{E}}_{e}$ and the induced polarization in the inclusion is shown by $\bar{P}$.

The electric field internal to the inclusion gives rise to a polarization density which is no longer necessarily aligned with the field because the material is non‐isotropic. The polarization density in the inclusion can be expressed as the tensor product of the permittivity contrast between the inclusion and the supporting medium and the (as yet unknown) internal field.

(12) $$\bar{P}=\left({\bar{\bar{\varepsilon }}}_{i}-{ɛ}_{e}\bar{\bar{1}}\right){\bar{E}}_{i}$$

For any ellipsoidal shape (including sphere, slab, and needle) with volume *V*, the polarization density throughout the particle is uniform and integrating over all space gives the field induced dipole moment of the inclusion, $\bar{p}$.

(13) $$\bar{p}=V\bar{P}=V\left({\bar{\bar{\varepsilon }}}_{i}-{ɛ}_{e}\bar{\bar{1}}\right){\bar{E}}_{i}$$

The dipole and the external field ( ${\bar{E}}_{e}$) are related by the polarizability tensor, $\bar{\bar{\alpha }}$.

(14) $$\bar{p}=\bar{\bar{\alpha }}\;{\bar{E}}_{e}$$

Equations (13) and (14) allow the determination of the polarizability, once the field internal to the inclusion has been expressed in terms of the shape of the inclusion and its permittivity. The polarization within the inclusion gives rise to a depolarization field ( ${\bar{E}}_{d}$), which depends on the shape of the inclusion through the symmetric and unit trace depolarization tensor, $\bar{\bar{L}}$.

(15) $${\bar{E}}_{d}=-\frac{1}{{ɛ}_{e}}\bar{\bar{L}}\cdot \bar{P}$$

The internal field is the sum of the external field and the depolarization field.

(16) $${\bar{E}}_{i}={\bar{E}}_{e}+{\bar{E}}_{d}$$

The depolarization tensor acts as a projection or screening operator describing the effect of the geometry of the inclusion on the depolarization field which results from its polarization. For instance, in the case of a needle, only polarization perpendicular to the needle axis contributes to the depolarizing field, whilst for a slab only polarization perpendicular to the slab face may contribute. Similarly for a sphere, all directions contribute and so the depolarization matrix is diagonal with a value of 1/3 for each diagonal element, as the trace of the depolarization tensor must be 1. It follows from eqs. (12), (15), and (16) that

(17) $${\bar{E}}_{i}={\bar{E}}_{e}-\frac{1}{{ɛ}_{e}}\bar{\bar{L}}\left({\bar{\bar{\varepsilon }}}_{i}-{ɛ}_{e}\bar{\bar{1}}\right){\bar{E}}_{i}$$

Rearrangement allows the internal field of the inclusion to be expressed in terms of the known permittivities, the shape of the inclusion and the external field.

(18) $$\begin{array}{c} {\bar{E}}_{i}\left({ɛ}_{e}\bar{\bar{1}}+\bar{\bar{L}}\cdot \left({\bar{\bar{\varepsilon }}}_{i}-{ɛ}_{e}\bar{\bar{1}}\right)\right)={ɛ}_{e}{\bar{E}}_{e}\\ {\bar{E}}_{i}={ɛ}_{e}{\bar{E}}_{e}{\left({ɛ}_{e}\bar{\bar{1}}+\bar{\bar{L}}\cdot \left({\bar{\bar{\varepsilon }}}_{i}-{ɛ}_{e}\bar{\bar{1}}\right)\right)}^{-1} \end{array}$$

Substituting the internal field expression eq. (17) into eq. (13) for the dipole moment and requiring the dipole moments calculated using the polarization density to equal those calculated from the polarizability allows the polarizability to be written

(19) $$\bar{\bar{\alpha }}=V{ɛ}_{e}\left({\bar{\bar{\varepsilon }}}_{i}-{ɛ}_{e}\bar{\bar{1}}\right){\left({ɛ}_{e}\bar{\bar{1}}+\bar{\bar{L}}\cdot \left({\bar{\bar{\varepsilon }}}_{i}-{ɛ}_{e}\bar{\bar{1}}\right)\right)}^{-1}$$

Although it has not been specified explicitly, the permittivity of the inclusion, and therefore the polarizability tensor are frequency dependent through the oscillator strengths of each phonon mode contributing to the permittivity according to eq. (10a). The calculation of the complex, frequency‐dependent polarizability tensor of the composite material is the key step in the calculation of its effective permittivity.

### The effective permittivity of a mixture

To extend this approach to include the effect of a number of inclusions we need to introduce the concept of an effective permittivity ( ${\bar{\bar{\varepsilon }}}_{\text{eff}}$), which describes the behavior of an average field, $〈\bar{E}〉$, where the angle brackets indicate an average over a volume of the composite material. It is required that the average electric flux density $〈\bar{D}〉$ is the same in the effective medium as in the composite medium

(20) $$〈\bar{D}〉={\bar{\bar{\varepsilon }}}_{\text{eff}}〈E〉={ɛ}_{e}〈\bar{E}〉+〈\bar{P}〉$$

The averaging is necessary because the polarization within a given inclusion has an effect on the field in other inclusions. The local field in the cavity left by a single inclusion embedded in the average polarization field is given by

(21) $${\bar{E}}_{L}=〈\bar{E}〉+\frac{1}{{ɛ}_{e}}\bar{\bar{L}}〈\bar{P}〉$$

The local field “excites” the inclusion resulting in a dipole moment $\bar{p}$ that is related to the polarization through the number density of inclusions (*n*) and through the polarizability of the inclusion, which is already known from eq. (19).

(22) $$〈\bar{P}〉=n{\bar{p}}_{}=n〈\bar{\bar{\alpha }}\;{\bar{E}}_{L}〉$$

The angle brackets around the product of the polarizability and the local field indicate that it is necessary to average the polarization according to the distribution of alignments of inclusions. In this work it will be assumed that the inclusions are randomly aligned. Substituting the expression for the local field [eq. (21)] gives

(23) $$〈\bar{P}〉={\left(\bar{\bar{1}}-\frac{n〈\bar{\bar{\alpha }}\;\bar{\bar{L}}〉}{{ɛ}_{e}}\right)}^{-1}n〈\bar{\bar{\alpha }}〉〈\bar{E}〉$$

### Mixing rules

There are many mixing rules which have been proposed to describe the homogenization of composite materials and a lot of work has been done in comparing their accuracy. Here two methods will be used. The first and the most commonly used method is the Maxwell‐Garnett mixing rule.^20^ Indeed this has been implied by the use of eq. (20) to define the effective permittivity. The other commonly used method is the Bruggeman mixing rule,^20^ which differs substantially in the way the two components of the system are treated. It is usually stated that the Maxwell‐Garnet mixing rule is good for low volume fractions of the inclusion and the Bruggeman approach should be better for higher volume fractions.^22^ In addition to these mixing rules one other approach will be described, namely the Averaged Permittivity (AP) mixing rule, which calculates the absorption spectrum ignoring the effects of the internal field on the absorption and can therefore be used as an indicator of the shifts in frequency and intensity which have occurred owing to the effect of particle shape.

### Maxwell‐Garnett mixing rule

The Maxwell‐Garnett approach for treating the properties of heterogeneous mixtures assumes that the average field and the average flux density result from volume fraction weighted sums. Substituting eq. (23) into eq. (20) gives the Maxwell‐Garnett effective permittivity;

(24) $${\bar{\bar{\varepsilon }}}_{\text{mg}}=\bar{\bar{1}}+{\left(\bar{\bar{1}}-\frac{n〈\bar{\bar{\alpha }}\;\;\bar{\bar{L}}〉}{{ɛ}_{e}}\right)}^{-1}n〈\bar{\bar{\alpha }}〉$$

The fact that the polarizability tensor has a volume term in it [eq. (19)] means that the terms in eq. (24) containing *n* $\bar{\bar{\alpha }}$ depend on the volume fraction *f*. Although written as a tensor, because the assumption has been made that the inclusions are randomly orientated, the effective permittivity has to be diagonal with equal complex values. Since the polarizability is complex and frequency dependent the effective permittivity and its calculation using eqs. (24) and (19) needs to be calculated over the frequency range of interest.

### Bruggeman mixing rule

In the Maxwell‐Garnett mixing formalism there is a distinction between the inclusion and the supporting medium which results in an asymmetry in the treatment of the two species in the mixture. Instead, the Bruggeman mixing rule assumes that each species is polarized against the background of the effective medium and therefore the polarization in the two components cancel each other out

(25) $$〈{\bar{P}}_{1}〉+〈{\bar{P}}_{2}〉=0$$

where the components are now labeled 1 and 2 rather than external and internal. The polarization for species 1 and 2 with a number density of species represented by *n*_1_ and *n*_2_ can be obtained from the polarizability of the species [eq. (22)]

(26) $$〈{\bar{P}}_{1}〉=n_{1}〈{\bar{\bar{\alpha }}}_{1}〉\bar{E}$$

Substituting eq. (26) into eq. (25) leads to the requirement that

(27) $$n_{1}〈{\bar{\bar{\alpha }}}_{1}〉+n_{2}〈{\bar{\bar{\alpha }}}_{2}〉=0$$

Taking eq. (19) and generalizing it for species *i*, (where *i* is 1 or 2) embedded in an effective permittivity given by ${\bar{\bar{\varepsilon }}}_{\text{br}}$

(28) $${\bar{\bar{\alpha }}}_{i}=V_{i}{\bar{\bar{\varepsilon }}}_{\text{br}}\left({\bar{\bar{\varepsilon }}}_{i}-{\bar{\bar{\varepsilon }}}_{\text{br}}\right){\left({\bar{\bar{\varepsilon }}}_{\text{br}}+\bar{\bar{L}}\cdot \left({\bar{\bar{\varepsilon }}}_{i}-{\bar{\bar{\varepsilon }}}_{\text{br}}\right)\right)}^{-1}$$

Equation (27) has to be solved for ${\bar{\bar{\varepsilon }}}_{\text{br}}$. Since the systems considered here are isotropic with random inclusions, a solution has to be found for a complex value of the Bruggeman permittivity at each frequency considered. An issue in the use of eq. (28) is that the same depolarization matrix is being used for both species, which is clearly not always appropriate. The solution of eq. (27) can be achieved either by iteration or by casting the equation as a minimization problem. The iterative approach implemented in PDielec involves repeated application of eq. (29) until convergence.^23^ The starting point for the iterations is taken as the Maxwell‐Garnett solution for the first frequency and then the solution at the previous frequency is used to start the iterations.

(29) $${\bar{\bar{\varepsilon }}}_{\text{br}}=\frac{f_{1}{\bar{\bar{\varepsilon }}}_{1}{\left[\bar{\bar{1}}+\bar{\bar{L}}\left({\bar{\bar{\varepsilon }}}_{1}-{\bar{\bar{\varepsilon }}}_{\text{br}}\right)\right]}^{-1}+f_{2}{\bar{\bar{\varepsilon }}}_{2}{\left[\bar{\bar{1}}+\bar{\bar{L}}\left({\bar{\bar{\varepsilon }}}_{2}-{\bar{\bar{\varepsilon }}}_{\text{br}}\right)\right]}^{-1}}{f_{1}{\left[\bar{\bar{1}}+\bar{\bar{L}}\left({\bar{\bar{\varepsilon }}}_{1}-{\bar{\bar{\varepsilon }}}_{\text{br}}\right)\right]}^{-1}+f_{2}{\left[\bar{\bar{1}}+\bar{\bar{L}}\left({\bar{\bar{\varepsilon }}}_{2}-{\bar{\bar{\varepsilon }}}_{\text{br}}\right)\right]}^{-1}}$$

Although the Bruggeman permittivity is written here as a tensor, the polarizabilities in eq. (27) have to be averaged over the random orientation of the inclusions and therefore the homogenized material is isotropic with a single complex value for the diagonal tensor. Also, as with the Maxwell‐Garnett mixing rule, since the polarizability is complex and frequency dependent, the effective permittivity is also, and its calculation using eq. (29) needs to be performed over the frequency range of interest.

The choice between using the Bruggeman or Maxwell‐Garnett model is often governed by the assumption that the Maxwell‐Garnett model works well at low concentrations and the Bruggeman model works better at higher concentrations. Work by Karkkainen *et al*. using a finite difference method for random mixtures of non‐absorbing materials indicated that the Bruggeman approximation works best when there is some clustering of the inclusions and the Maxwell Garnett model works best when there is no clustering.^24^

The Bruggeman solution has been shown to be unphysical in certain circumstances.^25^ In particular when the real components of the permittivities have different signs or when the absolute value of the real component is much larger than those of the imaginary component. Unfortunately, it may be that these conditions will apply to modeling infrared absorption. As a result only a few of the examples discussed below will include results using the Bruggeman mixing rule; the majority will use the Maxwell‐Garnett mixing rule.

### Averaged‐Permittivity mixing rule

It is useful to be able to compare the effective medium theories with the absorption predicted using no shape information, that is using only the TO frequencies.

(30) $${\bar{\bar{\varepsilon }}}_{\text{TO}}=f〈{\bar{\bar{\varepsilon }}}_{i}〉+\left(1-f\right){ɛ}_{e}$$

Equation (30) defines an isotropic permittivity which can be used to calculate such an absorption coefficient. The angle brackets indicate an average of orientation. This mixing rule provides a useful comparison between the absorption calculated without any shape effects and that calculated including shape effects using the other mixing rules presented above. At low concentrations the peak positions of the AP mixing rule will be at the TO frequencies.

## Implementation

The above theory has been implemented in a Python 2 package which is available for download.^26^ The package requires SCIPY,^27^ NUMPY,^27^ and if visualization of the predicted spectra is required MATPLOTLIB.^27^ At the moment the package has interfaces to two solid‐state QM codes, VASP^1^ and CASTEP.^2^ In addition, an interface is available for GULP^3^ which is a force field based solid‐state code. Examples of data sets for these packages are included with the distribution. The interface to these QM and MM codes reads information about the unit cell, the calculated normal modes, and the Born charge matrices; from these the permittivity is calculated over the frequency range requested. The absorption and molar absorption coefficients can be plotted along with the real and imaginary permittivities. Optionally all the information can be written to a comma separated values (csv) file for direct importing into a spreadsheet. The program is run from the command line. There are several command options and these are summarized below in Table 1. The command line must include the seedname for the files containing the CASTEP results or a directory name containing the VASP output from a calculation of the permittivity or the GULP output file name. Some of the options may be repeated. The package needs a shape to be specified (sphere, needle, plate, or ellipse). If no shape is specified on the command line a sphere is assumed.

**Table 1:** PDielec command line options.

Option	Default	Purpose	R^[a]^
‐method s	maxwell	The method is given by the string s and is either “ap,” “maxwell,” or “bruggeman.”	✓
‐sphere		The inclusion is a sphere, the default if no other shape is given.	
‐needle *h k l*		The inclusion is a needle whose unique direction is given by a the direction [*hkl*].	✓
‐plate *h k l*		The inclusion is a plate whose surface is defined by the Miller indices (*hkl*). Note that needles and ellipsoid use directions in crystal coordinates defined by [*hkl*]. For non‐orthogonal lattices the normal to the (*hkl*) is not necessarily the same as [*hkl*].	✓
‐ellipse *h k l z*		The inclusion is an ellipsoid, whose unique direction is given by [*hkl*], *z* specifies the eccentricity of the ellipsoid.	✓
‐vf *z*	0.1	*z* specifies the volume fraction	✓
‐mf *z*	0.0	*z* specifies a mass fraction from which the volume fraction is calculated. The calculation requires the density of the supporting matrix.	✓
‐matrix s	ptfe	The supporting matrix is defined by the string s. Options are “ptfe,” “kbr,” “nujol,” “air,” “vacuum,” “ldpe,” “mdpe,” “hdpe.” If the matrix is given in this way both the density and the permittivity of the supporting matrix are defined. Alternatively the ‐density and ‐dielectric options can be used.	
‐density *z*	2.2	*z* defines the density of the supporting matrix	
‐dielectric *z*	2.0	*z* defines the dielectric of the supporting matrix	
‐LO *h k l*		The frequencies corresponding to the longitudinal optic modes with a *k* vector direction (*h k l*) are calculated using eqs. (10) and (11)	✓
‐sigma *z*	5.0	*z* specifies the damping factor, σ, for all modes in cm^−1^, as used in eq. (10a)	
‐mode_sigma *k z*		The *k* ^th^ mode is assigned a specific *σ* (cm^−1^) given by *z*.	✓
‐*v*_min_ *z*	0.0	*z* is the starting wavenumber (cm^−1^) for the frequency range	
‐*v*_max_ *z*	300.0	*z* is the final wavenumber (cm^−1^) for the frequency range	
‐*i z*	0.2	*z* is the increment used to cover the frequency range (cm^−1^)	
‐plot s		Plot types are specified by the string s and they can be “absorption,” “molar_absorption,” “real,” or “imaginary”	✓
‐csv s		Output is sent to a comma separated file specified by the string s.	
‐print		Additional output is provided from the QM or MM calculation	
‐ignore *k*		Ignore the *k* ^th^ mode (any mode less than 5 cm^−1^ is ignored automatically)	✓
‐mode *k*		Only use the *k* ^th^ mode in the calculation of the permittivity	✓
‐optical *z*1 *z*2 *z*3		*z*1, *z*2, and *z*3 define the diagonal of the optical permittivity tensor	
‐optical_tensor *z*1 *z*2…*z*9		*z*1, *z*2 … *z*9 define the full optical permittivity tensor	

[a] This column indicates if a command line option can be used more than once.

The shape options: ellipse, slab, and needle, specify a unique axis [*hkl*] using the crystal axes of the unit cell. PDielec transforms these to a cartesian coordinate system using the unit cell lattice vectors. In the case of a slab morphology, the unique direction is normal to the surface specified by its Miller indices (*hkl*). The definitions of the various depolarization tensors are indicated in Table 2.

**Table 2:** Definitions used of the depolarization tensor.

Sphere	$\bar{\bar{L}}=\frac{1}{3}\left({\bar{V}}_{1}{\bar{V}}_{1}^{T}+{\bar{V}}_{2}{\bar{V}}_{2}^{T}+{\bar{V}}_{3}{\bar{V}}_{3}^{T}\right)$
Slab	$\bar{\bar{L}}={\bar{V}}_{1}{\bar{V}}_{1}^{T}$
Needle	$\bar{\bar{L}}=\frac{1}{2}\left({\bar{V}}_{2}{\bar{V}}_{2}^{T}+{\bar{V}}_{3}{\bar{V}}_{3}^{T}\right)$
Ellipsoid	$\bar{\bar{L}}=a{\bar{V}}_{1}{\bar{V}}_{1}^{T}+b{\bar{V}}_{2}{\bar{V}}_{2}^{T}+b{\bar{V}}_{3}{\bar{V}}_{3}^{T}$

The three directions defined by ${\bar{V}}_{1}$, ${\bar{V}}_{2}$, and ${\bar{V}}_{3}$ are mutually orthogonal cartesian vectors calculated from [*hkl*] for an ellipse, slab, or needle or (*hkl*) for a slab. In the case of a slab, needle or ellipsoid, ${\bar{V}}_{1}$ defines the unique direction and the other vectors are orthogonal to it. For the case of an ellipsoid, the parameters *a* and *b* in Table 2 depend on the ratio, *z*, of the length of unique axis length over the length of an axis perpendicular to it.^20^

For *z* > 1 the ellipsoid is prolate;

$e=\sqrt{1-z^{-2}},a=\frac{\left(1-e^{2}\right)}{2e^{3}}\left(\log\frac{1+e}{1-e}-2e\right),\;b=\frac{1}{2}\left(1-a\right)$

For *z* < 1 the ellipsoid is oblate;

$e=\sqrt{z^{-2}-1},a=\frac{\left(1+e^{2}\right)}{e^{3}}\left(e-\tan^{-1}e\right),\;b=\frac{1}{2}\left(1-a\right)$

From an experimental point of view it is often convenient to use a mass fraction rather than a volume fraction to indicate the amount of dielectrically active material present. PDielec allows mass fractions to be specified instead of a volume fraction, but this requires that the density of the supporting matrix is known. For convenience the package has a small database of the common supporting materials shown in Table 3. These can be specified through the ‐matrix option. In the case that the properties of the support material are different, the properties can be defined instead with the ‐dielectric and ‐density options.

**Table 3:** Physical properties of matrix materials in PDielec.

Name	Density	Permittivity	Description
ptfe	2.2	2.0	Polytetrafluorethylene
air	0.0	1.0	Air
vacuum	0.0	1.0	Vacuum
kbr	2.75	2.25	Potassium bromide
nujol	0.838	2.155	Nujol
hdpe	0.955	2.25	High density polyethylene
mdpe	0.933	2.25	Medium density polyethylene
ldpe	0.925	2.25	Low density polyethylene

The optical permittivity is normally calculated by the QM or MM program concerned. However, as this property reflects the electronic contribution to the permittivity at zero frequency, unless there is some treatment of electrons by the shell model, then in MM calculations the optical permittivity needs to be defined through the command line options ‐optical or ‐optical_tensor.

### Example command line uses of PDielec

pdielec ‐method ap ‐method maxwell \

‐sphere ‐plate 0 0 1 ‐needle 0 0 1 –LO 0 0 1.

This performs a calculation using the Averaged‐Permittivity and Maxwell‐Garnett mixing rules for spherical particles, plate‐like particles with a surface (001), and needle‐like particles with a unique direction lying along the [001] direction. The supporting matrix is taken to be PTFE and the default volume fraction (10%) is used. The results of a VASP calculation are stored in the current directory. There is no absorption output from this command as neither the ‐plot nor the ‐csv options were specified. The output includes the calculation of the LO modes along the (001) direction.

pdielec ‐vmin 300 ‐vmax 800 ‐sphere \

‐dielectric 3 ‐vf 0.1 ‐vf 0.2 ‐sigma 10 \

‐csv mgo.csv phonon

This performs a calculation for spherical particles varying the frequency from 300 to 800 cm^−1^, the permittivity of the supporting media is 3, two volume fractions are considered and a damping factor of 10 cm^−1^ is used. The results of a CASTEP calculation with the seed‐name “phonon” are analyzed and the results stored in mgo.csv for further analysis using a spreadsheet. In this example, a Maxwell‐Garnett mixing rule is used by default.

If visual inspection of the results is required then

pdielec ‐vmin 300 ‐vmax 800 ‐sphere \

‐dielectric 3 ‐vf 0.1 ‐vf 0.2 ‐sigma 10 \

‐csv mgo.csv \

‐plot molar_absorption phonon

will perform the same calculation but a graph showing the molar absorption coefficients will be displayed.

pdielec ‐matrix hdpe ‐method ap \

‐method maxwell \

‐sphere ‐plate −1 −1 −2 ‐vmax 2000 ‐mf 0.1 \

calcite.gout ‐csv calcite.csv

This command performs a calculation of the absorption spectrum resulting from a GULP calculation. The supporting matrix density and permittivity are those of high density polyethylene, the frequency range is 0–2000 cm^−1^, the mass fraction considered is 10%, the mixing rules used are Averaged‐Permittivity and Maxwell‐Garnett. Spheres and plates with the $\left(\bar{1}\bar{1}\bar{2}\right)$ surface are considered.

### Contents of the csv output file

If a csv output file is requested the file will contain the command used to perform the calculation. A brief summary is given of each active infrared mode, including the mode number, frequency, intensity, integrated molar absorption coefficient, its peak height (calculated from the intensity and damping factor), and the damping parameter used in the calculation. Following this is a table with a column for frequency followed by columns containing the real and imaginary permittivities, the absorption and molar absorption coefficients at each frequency.

## Examples

Several examples are given to illustrate applications of the package. The calculations used to provide the data for the permittivities are sufficiently accurate to illustrate aspects of the theory. The examples are chosen to show the package being used with the QM packages CASTEP and VASP and with the MM package GULP.

### MgO using CASTEP

Magnesium oxide is an isotropic medium, the initial unit cell and the space group symmetry (*Fm* $\bar{3}$ *m*) were taken from the Inorganic Crystal Structure Database (ICSD)^28^ reference number ICSD‐52026.^29^ The primitive cell was optimized using CASTEP. Norm‐conserving pseudo‐potentials were used to represent the core electrons of magnesium and oxygen. An energy cutoff of 1,000 eV was used with the PBE^30^ density functional and a *k*‐point spacing for the Monkhorst‐Pack grid of 0.04 Å^−1^. The primitive cell was optimized and a Density Functional Perturbation Theory (DFPT) calculation of the phonon spectrum at the gamma point was performed. The optimized lattice parameter was found to be 2.1234 Å, compared with the experimental value of 2.107 Å. Only three degenerate modes contribute to the permittivity. A summary of the results is presented in Table 4.

**Table 4:** Calculated properties of MgO.

Property	Values		Units
Unit cell dimensions^[a]^	2.123 (2.107)		Å
Space group	*Fm* $\bar{3}$ *m*		
Optical permittivity	3.14		
Static permittivity	10.0		
Phonon frequency (intensity)^[b]^	TO T 388.3 (9.29)	LO (001) 693.7	cm^−1^((D/Å)^2^/amu)

[a] The experimental values taken from Ref. 29 are given in brackets.

[b] The intensities are given in brackets, T indicates a triply degenerate mode.

**Figure 2:**
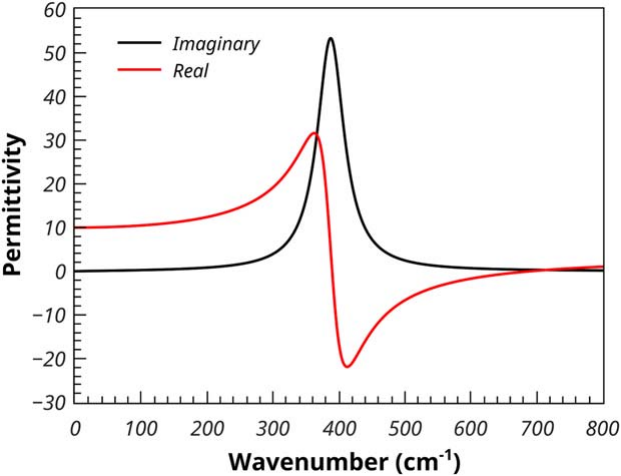
Calculated permittivity of MgO. [Color figure can be viewed in the online issue, which is available at wileyonlinelibrary.com.]

Because MgO is isotropic with only a single frequency contributing to the permittivity, it makes a useful example application to illustrate several features of PDielec. The real and imaginary frequency dependent permittivities are shown in Figure 2, where a damping factor (*σ*) of 10 cm^−1^ has been used. In the figure, the real permittivity at zero frequency corresponds to the static permittivity in Table 4, and at frequencies above the absorption at 388 cm^−1^ the permittivity tends to the optical permittivity as the frequency increases. The real permittivity has zero values at 388.3 and 693.7 cm^−1^ which are the TO and LO frequencies, respectively.

**Figure 3:**
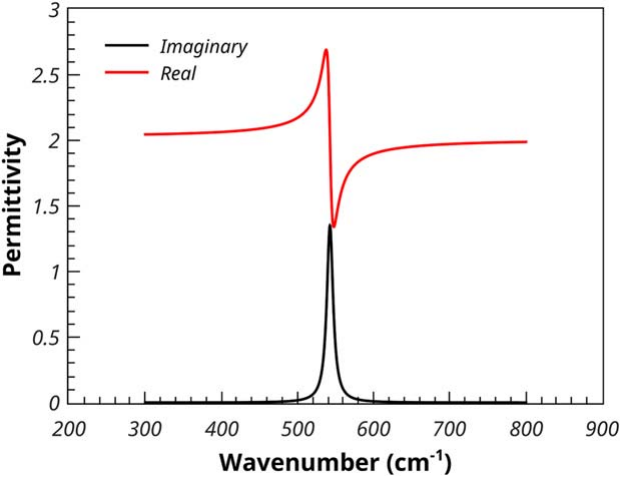
Calculated real and imaginary permittivities of a 10% volume fraction of MgO spheres in PTFE, calculated using the Maxwell‐Garnett method. [Color figure can be viewed in the online issue, which is available at wileyonlinelibrary.com.]

Using the Maxwell‐Garnett mixing rule, Figure 3 shows the calculated permittivities of a 10% volume fraction of MgO spheres in a supporting medium with a frequency independent permittivity of 2.0, which would be typical of a material such as PTFE. Due to the dilution effect, the real component has shifted to a base line value close to 2, and the absorption, as indicated by the maximum in the imaginary component has shifted by about 160 cm^−1^ to 550 cm^−1^.

**Figure 4:**
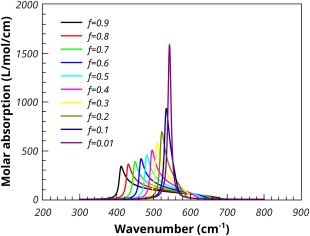
Effect of volume fraction on the Maxwell‐Garnett molar absorption coefficient of MgO spheres in PTFE. [Color figure can be viewed in the online issue, which is available at wileyonlinelibrary.com.]

The effect of volume fraction on the predicted molar absorption coefficient, using the Maxwell‐Garnett mixing rule, is shown in Figure 4. The lowest volume fraction of MgO gives the largest shift of the absorption peak to high frequency. As the volume fraction increases, the mixing rule predicts a broadening of the absorption, whilst the peak in the molar absorption coefficient moves to lower frequency. At the highest loading (*f* = 0.9) the maximum absorption occurs quite close to the TO frequency. The Maxwell‐Garnett mixing rule is regarded as being appropriate for low volume fractions and so should not be used for interpreting results in which higher volume fractions of absorbing media have been used.^20^

**Figure 5:**
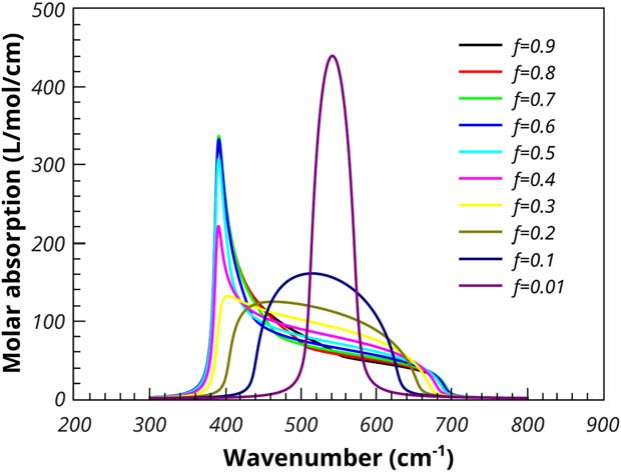
Effect of volume fraction on the Bruggeman molar absorption coefficient of MgO spheres in PTFE. [Color figure can be viewed in the online issue, which is available at wileyonlinelibrary.com.]

Figure 5 shows the same plot for the Bruggeman mixing rule. At low volume fractions, the Bruggeman mixing rule predicts a similar absorption to the Maxwell‐Garnett. Indeed as the volume fraction approaches zero the two rules predict the same absorption characteristics. However, even at the relatively low 1% loading, the Bruggeman mixing rule shows additional broadening of the peak, the shape of the absorption peak has lost its Lorentzian characteristic shape as can be seen clearly in Figure 5. At 10% loading the Bruggeman predicted absorption is broad with the peak shifted to lower wavenumber. This broadening increases with increased loading until, at the higher loadings, the TO peak begins to dominate the absorption.

**Figure 6:**
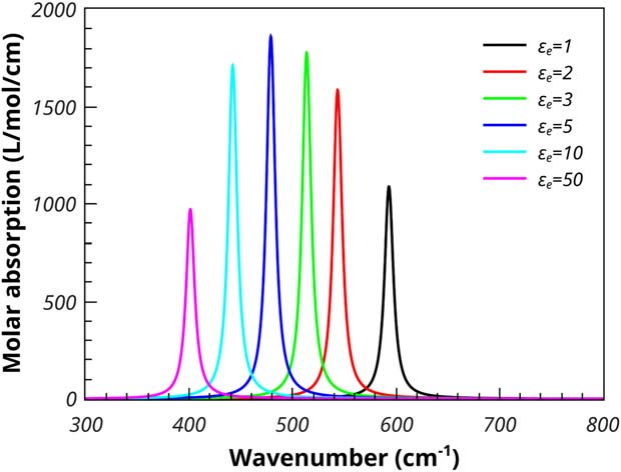
The Maxwell‐Garnett molar absorption coefficients of spherical MgO particles, 1% volume fraction, embedded in media of varying permittivities. [Color figure can be viewed in the online issue, which is available at wileyonlinelibrary.com.]

**Figure 7:**
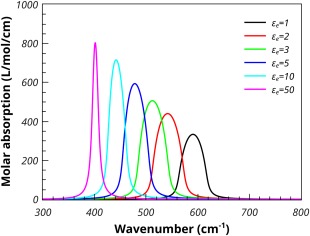
The Bruggeman molar absorption coefficients of spherical MgO particles, 1% volume fraction, embedded in media of varying permittivities. [Color figure can be viewed in the online issue, which is available at wileyonlinelibrary.com.]

Figure 6 shows the effect of varying the permittivity of the supporting medium. The calculations were performed on spherical MgO particles with a 1% volume fraction. The lowest permittivity is that of a vacuum (or air) and shows the highest shift of the absorption maximum to higher frequencies. Increasing the permittivity lowers the shift until it becomes quite small. A similar effect is seen for the Bruggeman mixing model. However, the absorption results for particles in a low dielectric medium is considerable broader than that seen in the Maxwell‐Garnet case. This broadening reduces as the permittivity of the medium increases (see Fig. 7).

### ZnO using VASP

**Figure 8:**
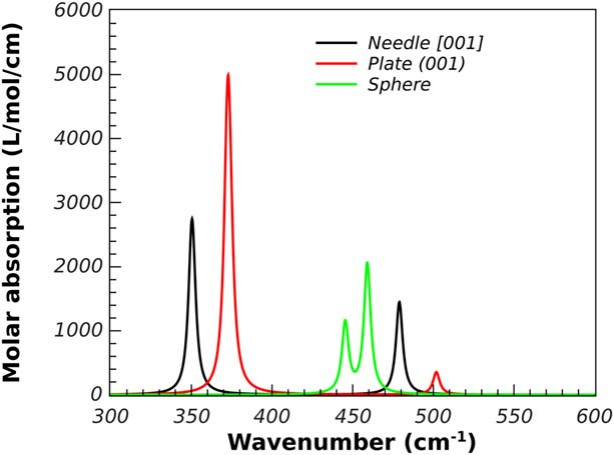
The effect of shape on the Maxwell‐Garnett molar absorption coefficient of 1% volume fraction ZnO in PTFE. [Color figure can be viewed in the online issue, which is available at wileyonlinelibrary.com.]

Zinc oxide crystallizes in space group *P6_3_mc* (wurtzite). All calculations were performed by VASP^1^ using projector augmented‐wave PAW^31^ pseudo‐potentials, the PBE^30^ density functional, an energy cutoff of 600 eV, and a *k*‐point resolution of approximately 0.1 Å^−1^. The initial unit cell was taken from the ICSD^28^ with code ICSD‐26170.^32^ The unit cell and atom positions were optimized using VASP and the permittivity was calculated using DFPT and the results reported in Table 5. Only two of the bands showed any significant intensity, a doubly degenerate band (E) with a TO frequency of 372.1 cm^−1^ and a non‐degenerate band (A) with a TO frequency of 350.0 cm^−1^. The LO frequency of the non‐degenerate band is shifted to 502.0 cm^−1^ for a wave‐vector with direction (001), whilst the degenerate modes are unaffected. In the case of the (010) direction the LO frequency of one of the E modes is shifted to 511.2 cm^−1^. It is known that ZnO can crystallize with a plate morphology^33^ with the (001) surface dominant. Calculations of the molar absorption were performed for a sphere, plate, and needle‐like shapes the unique direction of the plate and needle being the [001] directions. A volume fraction of 1% was chosen for these calculations and the predicted molar absorption coefficients for the Maxwell‐Garnett mixing rule is shown in Figure 8.

**Table 5:** Calculated properties of ZnO.

Property	Values			Units
Unit cell dimensions^[a]^	*a*,*b* = 3.295 (3.25) *c* = 5.285 (5.207)			Å
Space group	*P6_3_mc*			
Optical permittivity^[b]^	5.09, 5.09, 6.0			
Static permittivity^[b]^	10.83, 10.83, 11.67			
Phonon frequency^[c]^(intensity)	TO A 350.0 (17.1) E 372.1 (16.4)	LO (001) 502.0	LO (010) 511.2	cm^−1^ ((D/Å)^2^/amu)

[a] The experimental values taken from Ref. 32 are given in brackets.

[b] Only the diagonal components are given.

[c] The intensities are given in brackets, E and A indicate a doubly and non‐degenerate mode respectively.

For the Maxwell‐Garnett mixing rule, the sphere morphology results in the two absorption peaks shifting from their TO positions to higher wavenumber by about 80 cm^−1^. The plate morphology results in one of the peaks moving to higher wavenumber by about 130 cm^−1^, whilst the other remains at the TO position. The Maxwell‐Garnett results are in close accord with some experimental results by Yamamoto *et al*.^34^ who measured the infrared spectrum of ZnO smoke particles and observed peaks in the absorption at 380, 530, and 550 cm^−1^. Previous works^35^, ^36^ have also used effective medium theory to explain the observed spectrum.

### Calcite using GULP

Calcite is the most stable polymorph of calcium carbonate and the crystal structure belongs to the *R* $\bar{3}$ *c* space group. The force field and atomic structures used here are described in detail in work by Fisler et al.^37^ Briefly, the oxygen ions are described using a core‐shell model.^38^ The carbon–oxygen potential of the carbonate is taken to be a Morse potential and an additional three atom potential is used to maintain the O—C—O angle at 120°. The van der Waals interactions between non‐bonded atoms are taken to be Buckingham potentials and the charges on the calcium, carbon, and oxygen ions are +2, +1.3435, and −1.1145, respectively. The shell charge of the oxygen ion is −2.133 and the spring constant for the core–shell interaction is 52.74 eV/Å^2^.

The unit cell was optimized using the primitive unit cell and the full space group symmetry. The calculation of the phonon spectrum was performed without symmetry but still using the primitive cell of the lattice. A summary of the calculated properties is given in Table 6.

**Table 6:** Calculated properties of calcite.

Property	Values	Units
Primitive cell dimensions^[a]^	*a*,*b*,*c* = 6.376 (6.375)	Å
	*α*,*β*,*γ* = 46.0 (46.1)	Degrees
Space group	$R\bar{3}$ *c*	
Optical permittivity^[b]^	1.91, 1.91, 2.0	
Static permittivity^[b]^	6.7, 6.7, 7.1	
Phonon frequency^[c]^(intensity)	TO E 114.8 (2.39) A 127.4 (3.36) A 249.3 (1.23) E 320.7 (5.82) A 338.1 (4.14) E 620.1 (3.38) A 732.0 (26.89) E 1463.6 (16.97)	cm^−1^ ((D/Å)^2^/amu)

The experimental values taken from Ref. 37 are given in brackets.

Only the diagonal components are given.

The intensities are given in brackets, E and A indicate a doubly and non‐degenerate mode respectively.

**Figure 9:**
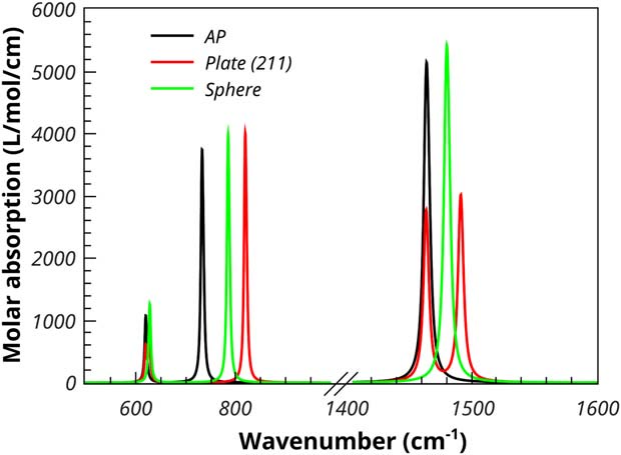
Calculated Maxwell‐Garnett absorption spectrum of 10% volume fraction of calcite in PTFE. [Color figure can be viewed in the online issue, which is available at wileyonlinelibrary.com.]

Figure 9 shows the results of analysis of the results using PDielec. The damping parameter used in the calculation was a value of 5 cm^−1^. A 10% volume fraction was used with sphere and plate morphologies for the particles. The unique axis for the plate was taken to be the normal to the (211) surfaces in the primitive cell axes (or the {104} surfaces in the standard unit cell). Such surfaces define the rhombohedral faces commonly seen in calcite crystals.^39^ Figure 9 shows that the doubly degenerate TO absorption peak at 620 cm^−1^ is not significantly affected by spherical particles and there is a small shift to higher frequencies in the case of plate‐like particles. The non‐degenerate TO transition at 732 cm^−1^, which corresponds to motion of the carbon atom of the carbonate along the unique direction of the slab, shows a shift to 786 and 819 cm^−1^ for the sphere and plate, respectively. The doubly degenerate peak at 1463 cm^−1^ is shifted to 1480 cm^−1^ by spherical particles and is split by plate‐like particles with one component which shifts to 1491 cm^−1^.

### Fluoroapatite using VASP

The line shapes of the infrared absorption of apatite and fluoroapatite were examined extensively by Balan *et al*.^16^ Their calculations included the effect of crystallite habit on the spectrum and the results reported here are similar to their conclusions. The method used by Balan *et al*. is an infinitely dilute Maxwell‐Garnett model, so the only difference between the methods used by them and those reported here using PDielec are the incorporation of the volume fraction into the theory and the use of an ellipsoidal shape for comparison with the other shapes.

**Figure 10:**
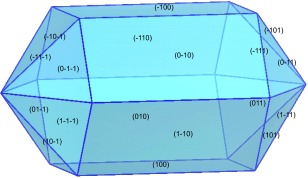
BDFH Morphology of fluoroapatite.

**Figure 11:**
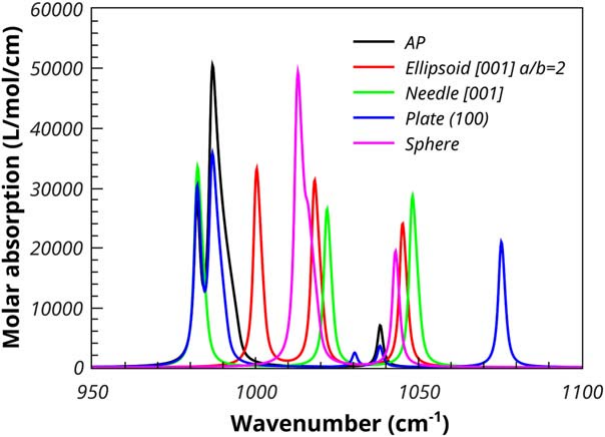
Calculated Maxwell‐Garnett absorption spectra of 10% fluoroapatite in PTFE. [Color figure can be viewed in the online issue, which is available at wileyonlinelibrary.com.]

All calculations were performed by VASP^1^ using projector augmented‐wave PAW^31^ pseudo‐potentials, the PBE^30^ density functional, an energy cutoff of 600 eV, and a *k*‐point resolution of approximately 0.1 Å^−1^. Table 7 summarizes the results of the calculations. Only the three highest frequency bands are reported and discussed. The TO intensity of the highest frequency band at 1038 cm^−1^ is low and will not be discussed further. The Bravais Friedel Donnay Harker (BFDH)^41^ crystal habit of the optimized crystal is shown in Figure 10. The habit was calculated using the Mercury software package.^42^ The BFDH crystal habit is often used to give an idea of the likely important faces of a crystal. It uses only the crystal lattice and space group to determine the crystal morphology. Figure 10 shows that the {100} surfaces form a tube which are capped by the {011} surfaces. The effect of different particle shapes on the predicted spectrum is shown in Figure 11. The calculations of the spectra were performed with a damping parameter (*σ*) of 2 cm^−1^. The ellipsoid was chosen to have an aspect ratio, *a*/*b*, of 2 and a principle axis along [001], which was compatible with the morphology predicted by the BDFH method. The two TO absorption frequencies at 981 and 986 cm^−1^ have A and E symmetry, respectively. Spherical crystallites result in three absorption peaks at around 1000, 1010, and 1015 cm^−1^. Needle‐shaped crystallites leave the A symmetry TO absorption peak at 981 cm^−1^ unaffected, but shift and split the E symmetry TO peak to 1020 and 1046 cm^−1^. A plate morphology with (100) surfaces results in the A and one component of the E TO absorption peak remaining at the TO frequencies, with the other component of the E shifting 85 cm^−1^ to 1075 cm^−1^. The ellipsoidal morphology show three shifted peaks at 1000, 1018, and 1045 cm^−1^. These results are consistent with those of Balan *et al*.,^16^ who gave detailed results for hydroxyapatite.

**Table 7:** Calculated properties of fluroapatite.

Property	Values	Units
Primitive cell dimensions^[a]^	*a*,*b* = 9.447 (9.417) *c* = 6.926 (6.875)	Å
Space group	$P6_{3}m$	
Optical permittivity^[b]^	2.891, 2.891, 2.894	
Static permittivity^[b]^	12.081, 12.081, 8.841	
Phonon frequency^[c]^(intensity)	TO A 981.8 (112.6) E 986.3 (101.0) E 1038.1 (7.92)	cm^−1^ ((D/Å)^2^/amu)

The experimental values taken from Ref. 40 are given in brackets.

Only the diagonal components are given.

The intensities are given in brackets, E and A indicate doubly and non‐degenerate modes, respectively.

### l‐Aspartic acid using CASTEP

l‐Aspartic acid is a zwitterion in the solid state and so the shape of the particles used in the measurement of IR and THz spectra maybe important. The starting geometry for optimization of the unit cell and molecular structure of l‐aspartic acid was taken from Derissen *et al*.^43^ The PBE^30^ functional was used with a plane wave energy cutoff of 1000 eV and norm conserving pseudo‐potentials. A dispersion correction using the Tkatchenko–Scheffler scheme^44^ available in CASTEP was applied for both the geometry optimization and the calculation of the phonon spectrum at the gamma point, with a value *S*_6_ scaling factor^44^ of 1.0. A summary of the results of the calculations is shown in Table 8.

**Table 8:** Calculated properties of l‐aspartic acid.

Property	Values	Units
Unit cell dimensions^[a]^	*a* = 7.597 (7.617)	Å
	*b* = 7.028 (6.982)	
	*c* = 5.113 (5.142)	
	*β* = 98.77 (99.84)	
Space group	$P2_{1}$	Degrees
Optical permittivity^[b]^	2.68, 2.20, 2.56	
Static permittivity^[b]^	4.58, 3.65, 3.65	
Phonon frequency^[c]^(intensity)	TO 84.5 (0.120) 104.7 (0.202) 106.0 (0.243) 115.3 (0.474) 137.3 (0.617) 1290.0 (55.0) 2945.9 (102.8) 2947.3 (48.2) 3053.7 (44.1)	cm^−1^ ((D/Å)^2^/amu)

[a] The experimental values taken from Ref. 43 are given in brackets.

[b] Only the diagonal components are given.

[c] Only selected transitions are tabulated. The intensities are given in brackets.

**Figure 12:**
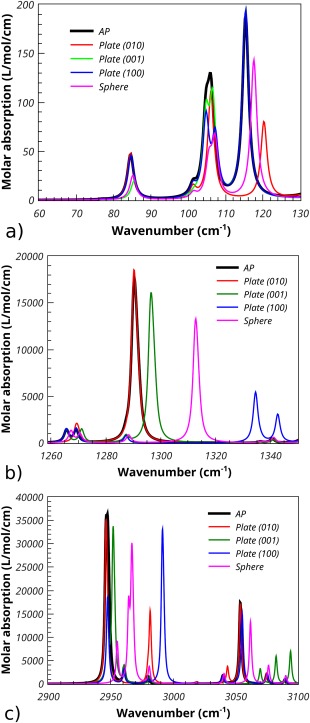
Calculated Maxwell‐Garnett absorption spectra of 10% volume fraction of l‐aspartic acid in PTFE. a) Frequency range 60–130 cm^−1^, b) Frequency range 1260–1340 cm^−1^, c) Frequency range 2900–3100 cm^−1^.

The THz spectrum of l‐aspartic acid has been reported by Juliano and Korter^45^ in the frequency range 0–90 cm^−1^. The infrared spectrum has been reported and assigned by Lopez Navarrete et al.^46^ Figure 12 shows the calculated absorption spectra for l‐aspartic acid for three frequency ranges. The calculation of the spectra used the Maxwell‐Garnett mixing rule with a 10% volume fraction of l‐aspartic acid in PTFE and for comparison the TO mixing rule. A damping factor of 2 cm^−1^ was used. Spherical and a variety of plate‐like inclusions were used to illustrate their effect on the absorption spectra. Figure 12a shows the frequency range from 60 to 130 cm^−1^ which is that covered by THz spectroscopy. The shifts observed for the different particle morphologies are not large, but the change in intensities is significant. The molecular motions associated with phonons at these frequencies tend to be whole molecule motion involving rotation. Figure 12b shows the frequency range from 1260 to 1340 cm^−1^. In this frequency range, bending of the carboxylate anion contributes to the spectrum significantly. The three different plate morphologies show different and significant shifts in the TO absorption peak at 1290 cm^−1^. The spherical morphology shows a shift of around 25 cm^−1^ to higher wavenumber. Figure 12c shows the spectra in the frequency range 2900–3100 cm^−1^, which corresponds to the motion of O—H (below 2980 cm^−1^) and N—H (above 2980 cm^−1^) stretching. The effect of the different possible crystal morphologies is large with shifts to higher frequency of up to 50 cm^−1^. The spectra below 3000 cm^−1^ arise from two TO absorptions at 2946 and 2947 cm^−1^. Because the motions associated with each mode interact differently with the internal field within each crystal they give rise to different shifts producing more complex spectra.

## Conclusions

The PDielec package has been described and examples given as to its application in calculating the infrared absorption spectrum of a dielectric material embedded in the supporting matrix. The shape of the crystallites can be taken into account by describing them as spheres, plates, needles, or ellipsoids. The package can calculate the dielectric response of the effective medium as well as the infrared absorption as a function of frequency. Several of the examples cover dielectric materials which have been well studied, both experimentally and theoretically and the results are in agreement with the previous work. The package is written in Python and can be extended relatively straightforwardly to interface with other packages. The results show the sensitivity of the absorption spectrum to the particle morphology and illustrate the complexity of interpreting IR and THz absorption spectra.

The PDielec package along with some example test cases for each QM or MM package supported is available on GitHub.^26^ The data used to create the figures and tables are openly available from the Leeds University data repository.^47^
